# Transgastric single-incision laparoscopic resection for a recurrent gastric adenoma at the prepyloric antrum: A case report

**DOI:** 10.1016/j.ijscr.2025.110940

**Published:** 2025-01-23

**Authors:** Xin Yu Zhuang, Can Wu, Jun Wen Wu, Xue Fei Yang, Zhong Hui Liu

**Affiliations:** Department of Surgery, The University of Hong Kong, The University of Hong Kong - Shenzhen Hospital, Shenzhen, China

**Keywords:** Gastric neoplasm, Laparoscopic surgery, Antrum, Pyloric

## Abstract

**Introduction:**

Endoscopic resection is suitable for most benign gastric or early stage cancerous polyps. Laparoscopic local resection is performed only for gastric polyps that are difficult to treat with endoscopic resection, such as recurrent or large polyps. However, when polyps are located in difficult regions, such as the gastric cardia and prepyloric antrum, wedge resection may damage the sphincter around the cardia or pylorus, resulting in postoperative deformity or stenosis.

**Presentation of case:**

A 66-year-old gentleman found a 2.5 cm recurrent adenoma at pre-pyloric antrum when he repeated a esophagogastoscopy 1 year after a 2 cm polyp removed by endoscopic mucosal resection (EMR) at the same site. Owing to submucosal fibrosis, neither EMR nor endoscopic submucosal dissection is considered suitable for recurrent adenoma because of the increased risk of perforation. Pyloric stenosis or deformity was expected with traditional laparoscopic wedge resection for such a lesion located at the pre-pyloric antrum. Instead, we successfully performed a transgastric single-incision laparoscopic en bloc resection of the adenoma. Precise dissection was performed during surgery. The patient's postoperative recovery was uneventful. A repeated esophagogasroscopy one year later showed no recurrence.

**Discussion:**

Transgastric single-incision laparoscopic resection for recurrent gastric mucosal lesions after previous endoscopic resection is technically feasible and safe.

**Conclusion:**

This procedure can be an alternative choice for local resection of recurrent benign gastric mucosal lesions, especially for those located in special regions such as the prepyloric antrum or gastric cardia.

## Highlights

• Recurrent gastric adenomas after endoscopic resection usually require surgical treatment.
• Pyloric stenosis or deformity was expected with traditional surgery for lesions located at the pre-pyloric antrum.
• Surgery was successfully performed with good preservation of the pylorus.

## 1 Introduction

Local recurrence of benign gastric polyps after endoscopic mucosal resection (EMR) generally renders endoscopic surgery unsuitable owing to the increased risk of perforation caused by postoperative submucosal fibrosis. Surgical resection is typically required in such cases. Laparoscopic or open local resection is the standard surgical procedure for the treatment of recurrent polyps. However, it is difficult to accurately locate mucosal lesions and perform precise resections during laparoscopic or open surgery. If the lesion is located in a special region, such as the pericardia or prepyloric antrum, precise dissection without injury to the cardia or pylorus is necessary to prevent postoperative stenosis or deformity. Transgastric laparoscopic surgery can be used to perform precise intragastric dissection of benign gastric lesions under laparoscopic visualization [1,2] and therefore may be a good alternative for the treatment of benign gastric lesions located around the cardia or in the prepyloric antrum. In this study, we present a case of a recurrent gastric adenoma in the prepyloric antrum that was successfully treated with transgastric single-incision laparoscopic resection. The paper's use of cases adheres to the requirements of The SCARE Guidelines [3].

## 2 Presentation of case

**Fig. 1:**
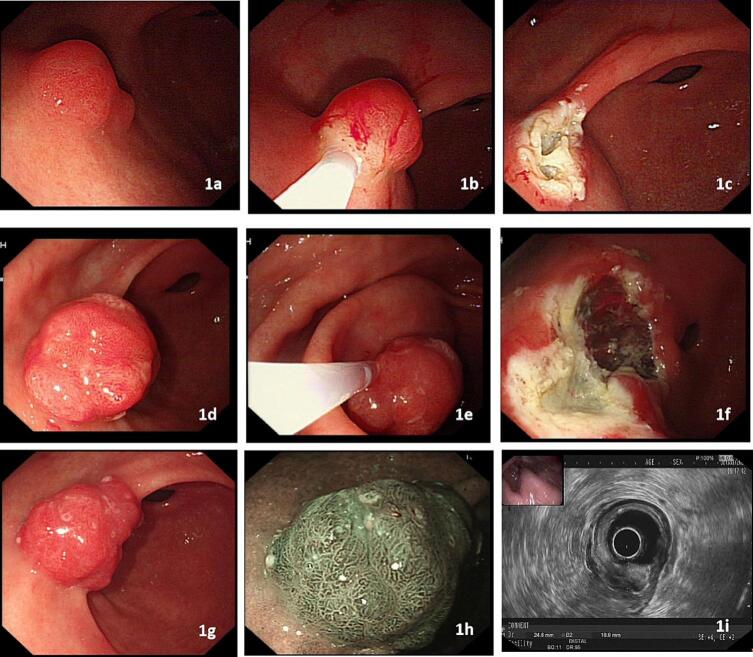
a, b, c. A 1.5 cm prepyloric polyp was removed via endoscopic mucosal resection (EMR); d, e, f. a recurrent 2 cm polyp was found on esophagogastroscopy 1 year later. An EMR of the recurrent polyp was performed; g, h, i. another 2.5 cm polypoid lesion was found again via esophagogastroscopy 1 year later at the same site. EUS indicated a mucosal lesion with severe submucosal fibrosis.

A 66-year-old man underwent EMR of a 1.5 cm prepyloric polyp (Fig. 1a, b, c). Pathological examination revealed a hyperplastic polyp. A recurrent 2 cm polyp was found on esophagogastroscopy 1 year later. EMR of the recurrent polyp was performed, and pathology revealed a foveolar-type adenoma with low-grade dysplasia (Fig. 1d, e, f), margin was close to the lesion. Another 2.5 cm polypoid lesion was found via esophagogastroscopy 1 year later at the same site (Fig. 1g, h, i). In view of submucosal fibrosis, EMR or endoscopic submucosal dissection (ESD) for recurrent polyps is not considered suitable because of the increased risk of perforation. Pyloric stenosis or deformity was expected after traditional laparoscopic wedge resection for lesions located in the prepyloric antrum. Instead, we successfully performed a transgastric single-incision laparoscopic en bloc resection of the lesion.

**Fig. 2:**
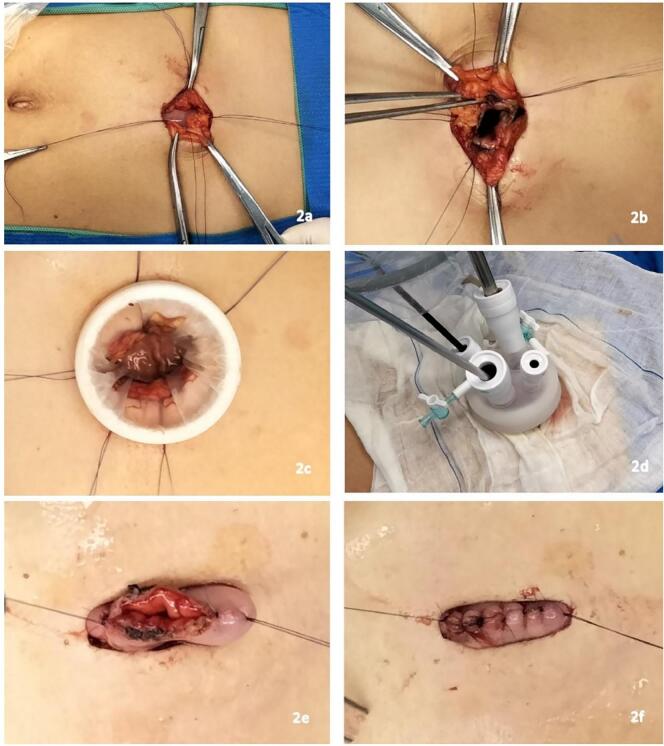
a. The distended stomach underneath the wound was sutured to the abdominal wall with four absorbable sutures; b, c, d. a 2.5 cm longitudinal incision at the anterior gastric wall was made, and a single-port device with four trocars (two 12 mm and two 5 mm) was inserted into the gastric cavity; e, f. the anterior gastric incision was closed with interrupted suture after completion of the laparoscopic procedure.

**Fig. 3:**
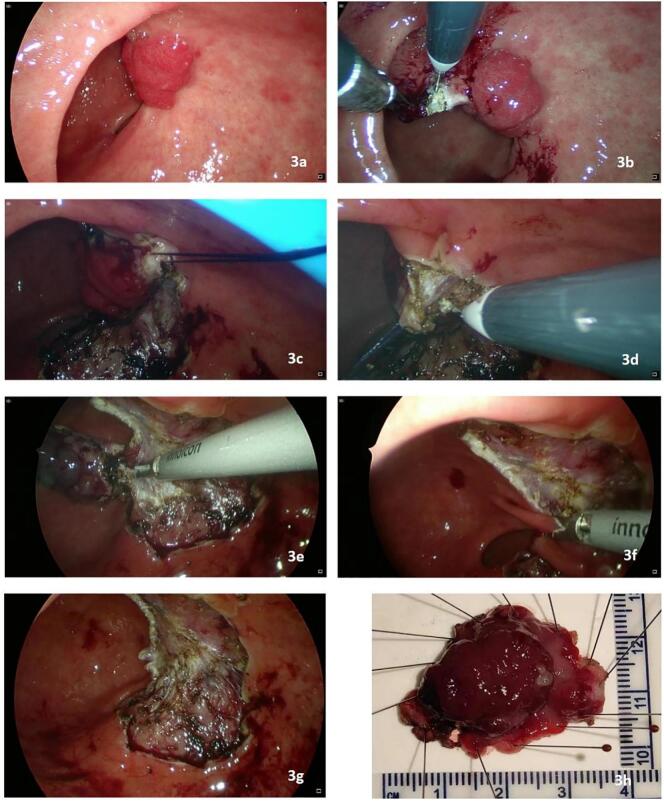
a. Intragastric laparoscopy showing a well-visualized mucosal legion in the prepyloric region; b. the mucosa was cut open 5 mm from the edge of the polyp with an electric hook; c, d. a stitch was made in the normal mucosa near the polyp to aid in exposure by lifting during submucosal dissection; e. an ultrasonic dissector was used for dissection when the lifting tension was insufficient; f. the peri-pyloric mucosa was preserved during dissection to prevent postoperative pyloric deformation or stenosis; g. the gastric defect after dissection was intact without perforation. It was not sutured to avoid stretching of the gastric wall and potentially causing pyloric deformation; h. the size of the specimen was 25 mm × 20 mm, and the gross margin was clear.

After induction of general anesthesia, the patient was placed in the supine split-leg position. A small vertical incision was made in the upper midline region after a gastroscope was inserted into the stomach, and CO2 was inflated into the stomach to distend it such that the anterior gastric wall was in close proximity to the anterior abdominal wall. The distended stomach underneath the wound was sutured to the abdominal wall using four absorbable sutures (Fig. 2a). A 2.5 cm longitudinal incision was made at the anterior gastric wall, and a single-port device with four trocars (two 12 mm and two 5 mm) was inserted into the gastric cavity (Fig. 2b, c, d). Gastric distension was maintained through CO2 insufflation, with the pressure regulated at 10 mmHg. The mucosal lesion was well-visualized in the prepyloric region (Fig. 3a), 5 mm from the edge of the polyp, and the mucosa was cut with an electric hook (Fig. 3b). Because the polyp pathology was benign, only the mucosa and submucosa were dissected to preserve the integrity of the muscularis propria. A stitch was placed in the normal mucosa near the polyp, and suture lifting was employed to aid in mucosal exposure during dissection (Fig. 3c, d). An ultrasonic dissector was used when lifting tension was insufficient (Fig. 3e). Care was taken to protect the peripyloric mucosa during dissection to prevent postoperative pyloric deformation or stenosis (Fig. 3f). After complete dissection, the lesion was completely excised through an incision. The gastric wall wound was observed to be intact without perforation, and suturing was avoided to prevent pylorus traction and deformation (Fig. 3g). The anterior gastric incision was closed with interrupted sutures using 4–0 Mathon sutures (Fig. 2e, f). The dimensions of the specimens were 25 × 20 mm (Fig. 3h). The operative time was 2 h. The patient resumed a liquid diet 2 days after the operation and was discharged on postoperative day 4. Pathology revealed that the polyp was hamartomatous, and the resection margin was clear. The patient repeated a esophagogastroscopy one year later, showing that the gastric antrum wound healed well without recurrence.

## 3 Discussion

Gastric polyps are elevated lesions that protrude from the gastric mucosa and are classified as neoplastic, hyperplastic, inflammatory, hamartomatous, developmental, mesenchymal, or miscellaneous [4]. Statistically, the use of upper gastrointestinal endoscopy has increased the detection rate of gastric polyps to 6% [5]. Endoscopic polyp appearance cannot differentiate histologic subtypes; therefore biopsy or polypectomy is recommended when a polyp is encountered [6], and some authors recommend polypectomy of all lesions >0.5 cm [7]. EMR is a recommended and preferred method for treating benign or early cancerous gastric mucosal lesion in diameter of 10-15 mm [8]. EMR enables successful treatment of most benign gastric polyps; however, EMR technology has a local recurrence rate of approximately 9 %–18% [8,9].

Endoscopic resection is suitable for most benign gastric or early stage cancerous polyps. Laparoscopic local resection is performed only for gastric polyps that are difficult to treat with endoscopic resection, such as recurrent or large polyps. However, when polyps are located in difficult regions, such as the gastric cardia and prepyloric antrum, wedge resection may damage the sphincter around the cardia or pylorus, resulting in postoperative deformity or stenosis [10]; therefore, some patients may have to undergo gastrectomy, which has a much greater rate of postoperative complications and a lower quality of life.

In this study, we introduced a novel minimally invasive surgery for the local resection of a recurrent benign gastric mucosal lesion located in a challenging anatomical region. This technique offers several advantages. First, the procedure was uncomplicated; the distended stomach underneath the wound was sutured to the abdominal wall, and the single-port device could be easily inserted into the gastric cavity after an incision of the anterior gastric body was made. Second, the surgical procedure was performed under laparoscopic visualization, which allowed precise tumor dissection and good protection of the normal anatomy of the pylorus from injury. Third, only one small skin incision was made using this procedure. The specimens were retrieved through a single incision. Cosmetic results are generally better than those of traditional laparoscopic resection. Fourth, conversion to laparoscopic or open surgery is easy when necessary. When converting to laparoscopic surgery, a single-incision trocar can be maintained, and other operating trocars can be added. During conversion to open surgery, a single-incision wound can be readily enlarged.

There may be some drawbacks associated with this surgical procedure. First, the gastric wall incision is directly connected to the abdominal wall, which may increase the risk of abdominal wall wound infection. Second, it is a single-port laparoscopic surgery which requires high surgical skills of the surgeon, and must be performed by experienced surgeons especially with an enough skill of hand-sew in narrow space; Third, since the tumor is removed through the abdominal wall incision, it is best to remove it through the specimen bag to reduce the risk of port-site implantation and recurrence. Finally, the procedure is not suitable for lesions in the anterior wall of the gastric body, whether early cancer or submucosal tumors, for the single-port trocar is assigned on the anterior wall of the gastric body, which will limit the manipulation angle of the instruments.

## 4 Conclusion

Transgastric single-incision laparoscopic surgery can serve as an alternative option for local resection of recurrent benign or early cancerous gastric mucosal lesions, especially those located in special regions such as the prepyloric antrum or gastric cardia. It may enable good preservation of the normal anatomy of the pylorus or cardia.

## Consent

Written informed consent was obtained from the patient for publication and any accompanying images. A copy of the written consent is available for review by the Editor-in-Chief of this journal on request.

## Ethical approval

The study was approved by the Hospital Ethics Committee (IRB number: [2024]354).

## Guarantor

Zhong Hui Liu

## Research registration number

This is not the first surgical procedure used in humans.

## Funding

This study was supported by the 10.13039/501100003785Guangdong Medical Research Foundation of China, grant number A2020603. The sponsor provided funding to support this study but was not involved in the content of the study.

## Author contribution

Xin Yu Zhuang^1^, M.D., Essay writing and organization

Can Wu^1^, M.D., To perform this operation

Jun Wen Wu^1^, M.D., To perform this operation

Xue Fei Yang^1^, M.D.; Ph.D., Revise and proofread articles

Zhong Hui Liu^1^, M.D.; Ph.D., Operation and article correction and proofreading

## Conflict of interest statement

The authors declare that they have no conflicts of interest.
